# Data on treatment of sewage wastewater by electrocoagulation using punched aluminum electrode and characterization of generated sludge

**DOI:** 10.1016/j.dib.2018.04.020

**Published:** 2018-04-11

**Authors:** Vinita Khandegar, Sanigdha Acharya, Arinjay K. Jain

**Affiliations:** University School of Chemical Technology, Guru Gobind Singh Indraprastha University, Dwarka, New Delhi 110078, India

**Keywords:** Electrocoagulation, Sewage wastewater, Aluminum, Plane, Punched electrode

## Abstract

The electrocoagulation setup must be optimized in order to design an economically feasible process. Therefore, in this work, the effect of the punched aluminum electrode on the performance of the electrocoagulation (EC) has been investigated. A series of experiments were performed for treatment of sewage wastewater using plane electrode and compare with punched electrodes. Effect of contact time, voltage, electrode spacing and stirring speed has been optimized for removal of Biochemical oxygen demand (BOD) and Total dissolved solids (TDS). It was observed that the performance of electrocoagulation process increased using punched electrode. Also, the less operating cost noticed in punched electrode as compared to a plane electrode for (70–80%) removal of BOD and TDS. These data would be useful in designing of an EC reactor to obtain high removal efficiency at low energy consumption.

## Abbreviations and nomenclature

*a*: Cost of electricity/kWh = 0.08 USD/kWh
*b*: Cost of electrode/kg electrode = 1.77 USD/kg Al
BOD: Biochemical oxygen demand (mg/L)
*C*_f_: Final concentrations of BOD and TSS (mg/)
*C*_o_: Initial concentrations of BOD and TSS (mg/L)
EC: Electrocoagulation
ELC: Electrode consumption (kg of electrode/m^3^of effluent)
ENC: Energy consumption (kWh/m^3^of effluent)
*F*: Faraday's constant = 96, 487 (C/mole)
*I*: Applied current (A)
*M*: Relative molar mass of the electrode = 26.98 (g/mole)
*n*: Number of electrons in oxidation/reduction reaction = 3
OP: Operating cost (USD/ m^3^of effluent)
*ɸ*: Diameter (mm) = 5 mm
*t*: Electrolysis time (h or s)
TDS: Total dissolved solids (mg/L)
*U*: Applied voltage (V)
*V*: Volume of treated effluent (m^3^)

**Specifications table**

**Table:** 

Subject area	Environment and sewage treatment
More specific subject area	Environmental Science
Type of data	Table and Figure
How data was acquired	Water analysis kit via NPC363D, India
Data format	Raw, analyzed
Experimental factor	– Sewage water was collected from MGD Waste Water Treatment Plant at Pappankalan, New Delhi, India. Characterization of wastewater is shown in Table 1.
	– 1 L glass beaker was used with an 800 mL working volume
	– Effect of contact time, voltage, electrode spacing and stirring speed were investigated. The detailed operating conditions are given in Table 2
	– Effects of different aluminum electrode configurations such as plane and punched (1, 2, 3 and 4) with ɸ 5 mm diameter was used to investigate the effect of electrode shape on operating cost of the electrocoagulation. A detailed comparison is given in Table 3.
Experimental features	– Electrocoagulation is a versatile technique used for treating various types of industrial effluent. The shape of the electrodes and operating cost are crucial in the electrocoagulation process.
Data source location	New Delhi, India
Data accessibility	This article contains all the dataset

**Value of the data**

• The effect of the punched aluminum electrode and operating cost of the electrocoagulation (EC) has been investigated.
• >95% BOD and TDS have been removed from sewage wastewater using punched electrodes. This dataset will be useful for designing of an economically feasible process in wastewater treatment area.
• This dataset showed that the less operating cost required for complete removal of pollutant by applying punched electrode as compare to the plane electrode.
• From the environmental esthetic point of view, the utilization and disposal of electrocoagulation generated sludge are very important. Therefore characterization of floc generated by EC has been done for further use.

### 1 Data

**Fig. 1:**
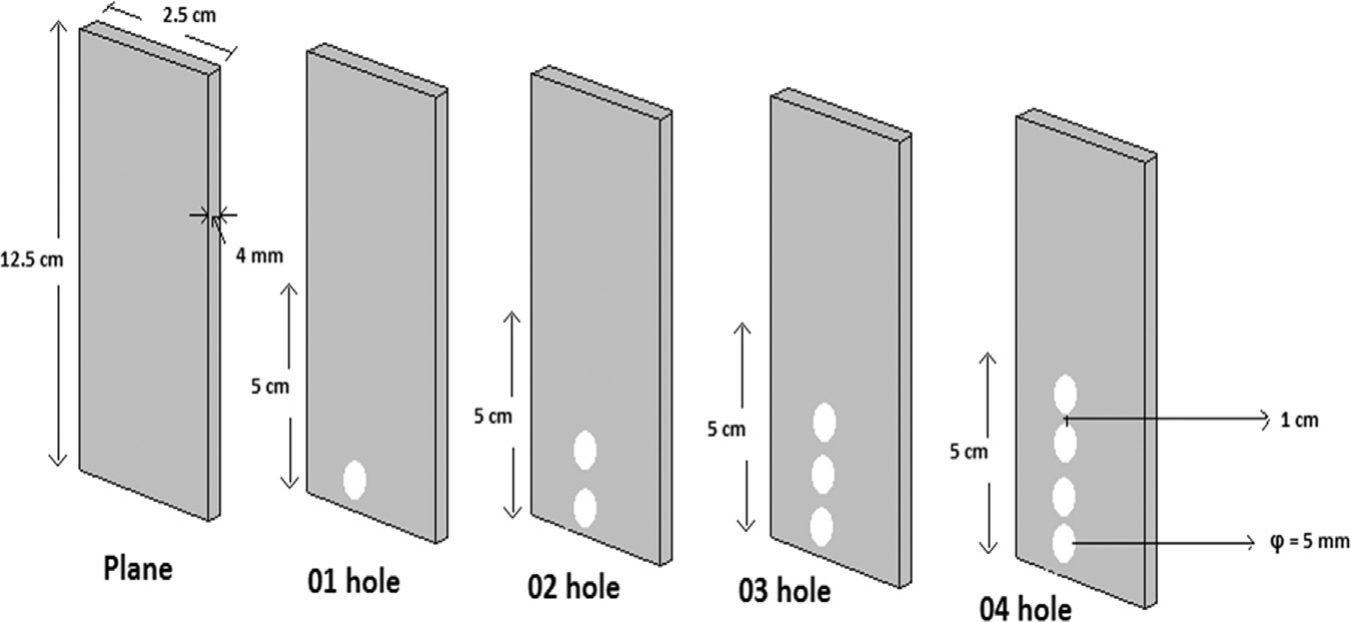
Geometry of the electrodes.

**Fig. 2:**
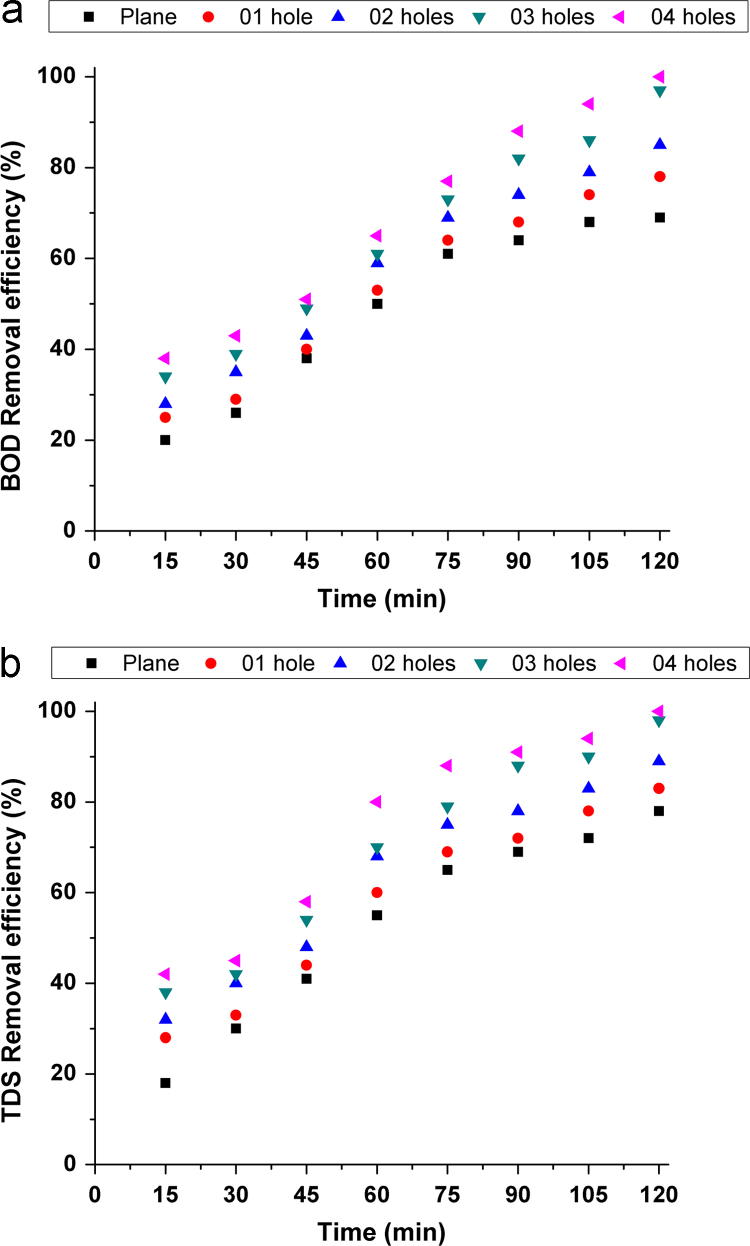
Effect of time on removal efficiency (a) BOD (b) TDS.

**Fig. 3:**
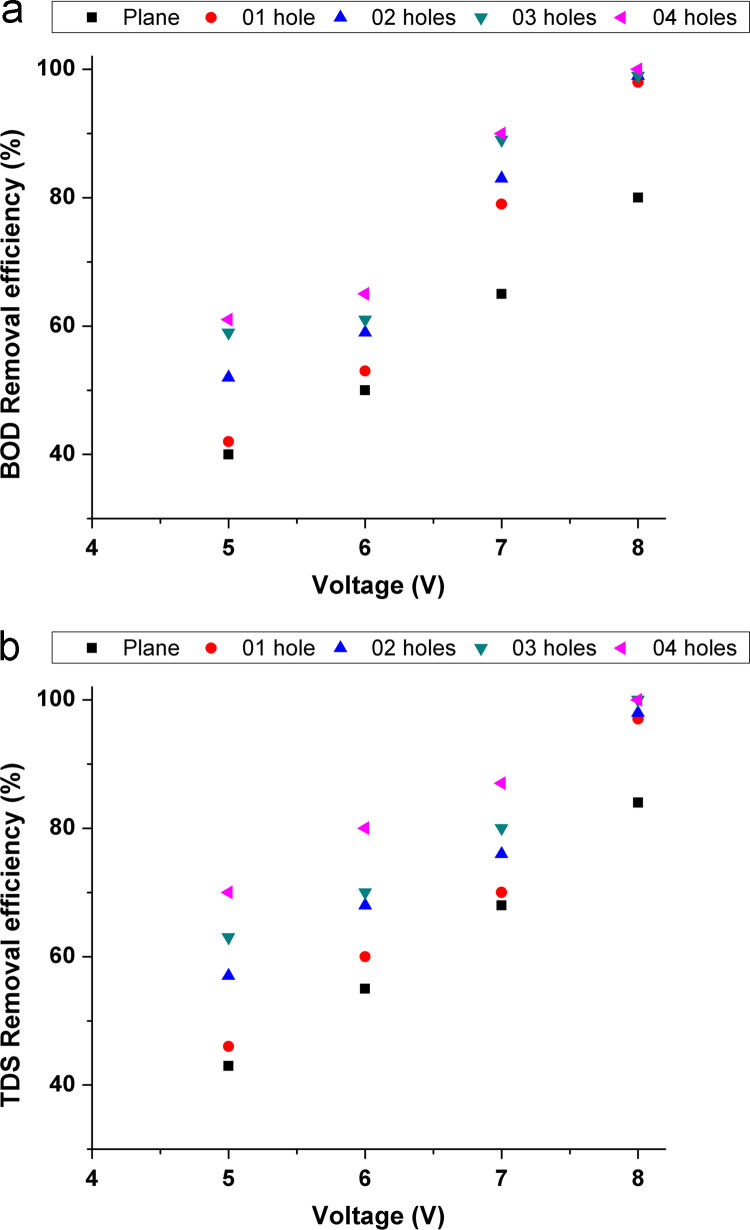
Effect of voltage on removal efficiency (a) BOD (b) TDS.

**Fig. 4:**
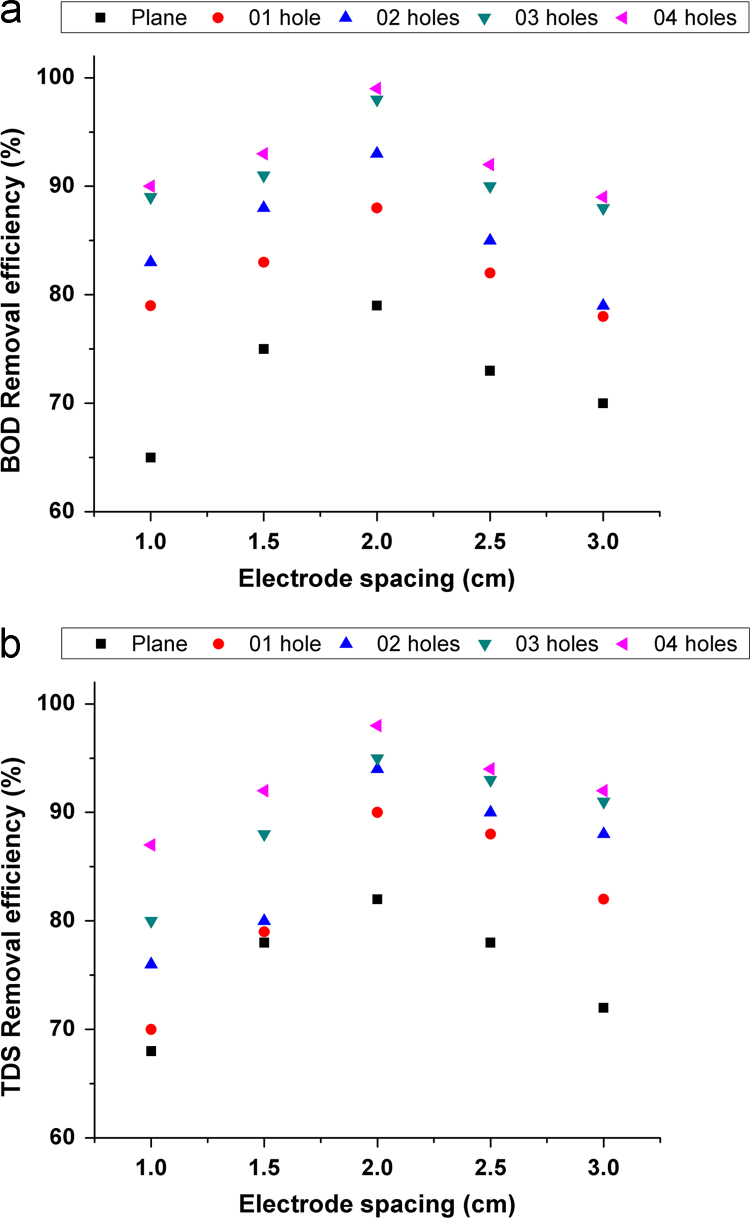
Effect of electrode spacing on removal efficiency (a) BOD (b) TDS.

**Fig. 5:**
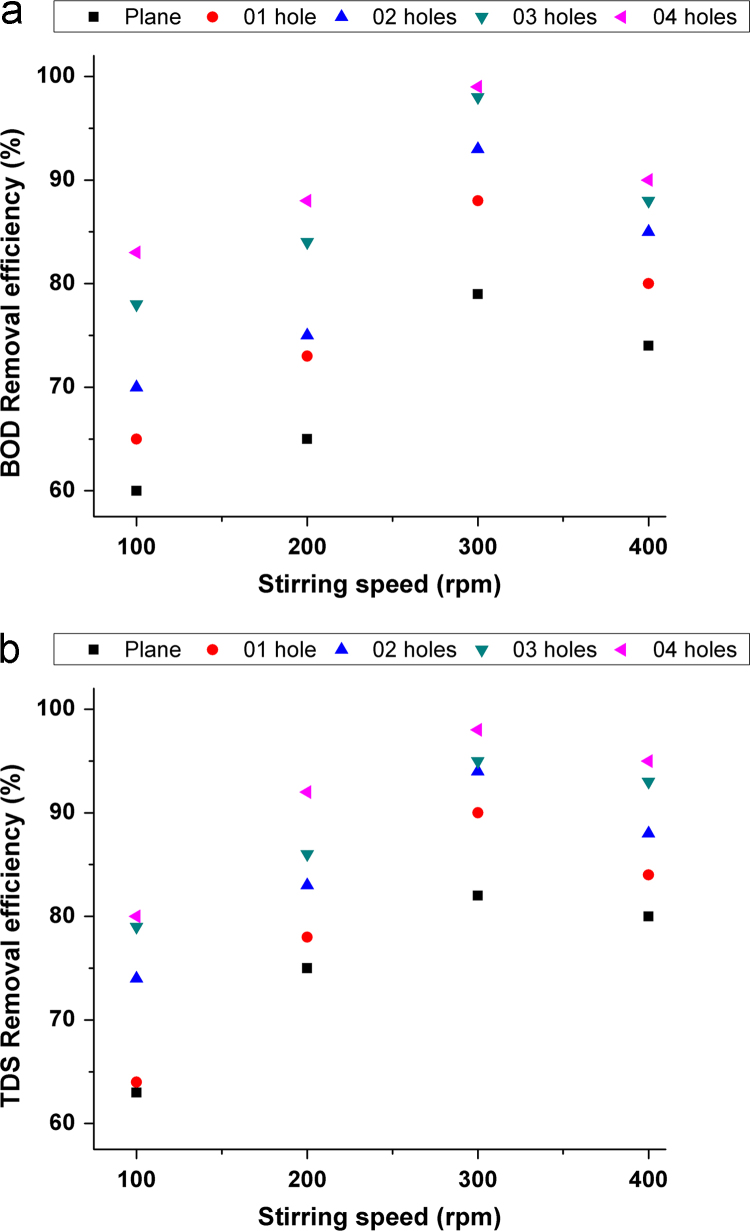
Effect of stirring speed on removal efficiency (a) BOD (b) TDS.

**Fig. 6:**
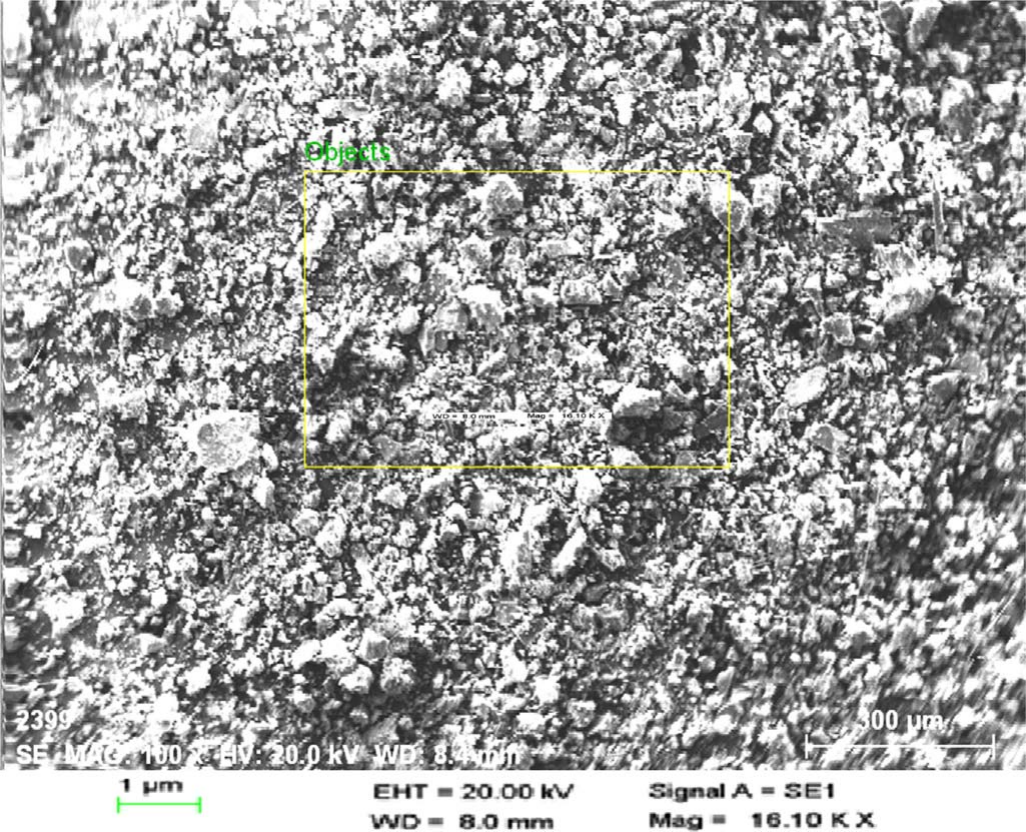
Image of SEM.

**Fig. 7:**
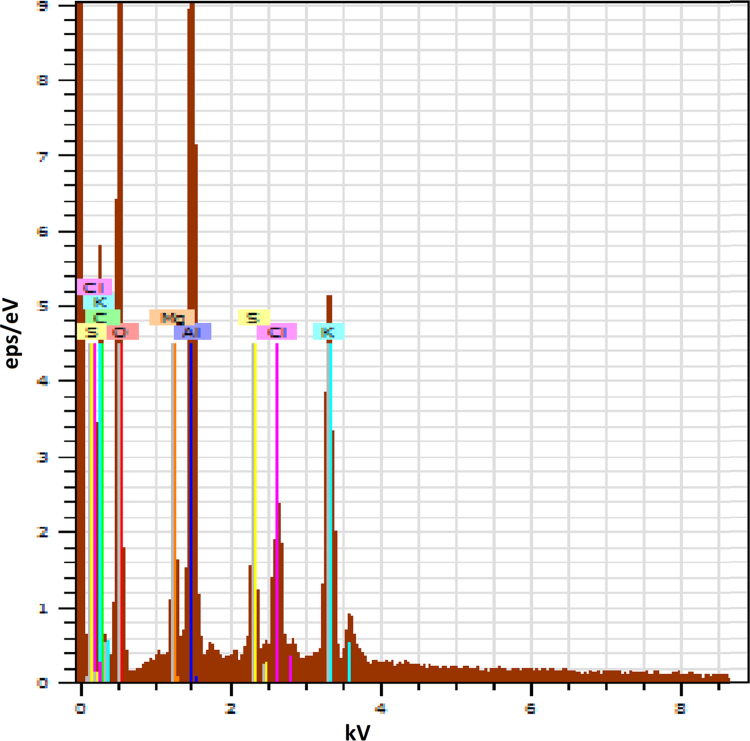
Image of EDX.

This dataset contains 3 Tables and 7 Figures that represent the performance evaluation of electrocoagulation process using punched electrodes and the plane electrode. The characteristics of collected sewage sample are presented in Table 1. Details of operating parameters are given in Table 2. The different electrode configurations are shown in Fig. 1. The dataset in order to optimize the effect of contact time, voltage, electrode spacing and stirring speed on the removal of BOD and TDS from sewage wastewater is given in Fig. 2, Fig. 3, Fig. 4, Fig. 5. The energy consumption and operating cost are demonstrated in Table 3. After electrocoagulation, the sludge was characterized by scanning electron microscopic (SEM EVO 50), energy-dispersive X-ray spectroscopy (EDAX, model Penta FET Precision) and the results are shown Fig. 6, Fig. 7 respectively.

**Table 1:** Characterization of sewage wastewater.

**Parameter**	**Studied sample**
pH	7.19
Color	Blackish
Turbidity (NTU)	203
Total dissolved solids (mg/L)	**910**
Total suspended solids (mg/L)	130
Conductivity (mS/cm)	34.48
Salinity (mg/L)	1267
BOD (mg/L)	**69.22**
COD (mg/L)	231.8
Oil & grease (mg/L)	Nil
Nitrates (mg/L)	Nil
Sulphate (mg/L)	78.12
Phosphate (mg/L)	38.12

**Table 2:** Operating condition for treatment of sewage water.

**Type of experiment**	**Contact time (min)**	**Voltage (V)**	**Electrode spacing (cm)**	**Stirring speed (rpm)**	**BOD concentration (mg/L)**	**TSS concentration (mg/L)**
Effect of contact time	15–120	5	1	300	69.22	910
Effect of voltage	60	5–8	1	300	69.22	910
Effect of electrode spacing	60	7	1–3	300	69.22	910
Effect of stirring speed	60	7	2	100–400	69.22	910

### 2 Experimental design, materials, and methods

#### 2.1 Sample collection

Sewage wastewater was collected from MGD Waste Water Treatment Plant at Pappankalan, New Delhi, India and characterized for various parameters using Water analysis kit (NPC363D, India) (see in Table 1).

#### 2.2 Experimental procedure

Experiments were conducted in a 1 L glass beaker in the batch mode of operation using aluminum electrode pair (12.5 cm × 2.5 cm × 0.4 cm). The electrode pair was immersed in wastewater to a depth of 5 cm with the electrodes around 1 cm apart. The effective area of the electrode pair was 12.2 cm^2^. The assembly was connected to a direct current power source (Science tech 4074, India). The experimental setup was similar to provided in our previous studies [1], [2]. Experiments were carried out using aluminum electrodes with/without punched holes (see in Fig. 1) to study the effect of electrode configuration on the performance of electrocoagulation. Various operating parameters used in EC experiments (see in Table 2). The removal efficiency of BOD and TDS from sewage water was investigated after each experiment using Eq. (1) and results are shown in Fig. 2, Fig. 3, Fig. 4, Fig. 5. To observe the energy consumption and operating cost throughout the experiments following equations were adapted from literature [3], [4], [5], [6], [7] and the results are tabulated in Table 3.

(1) $$Removal\;efficiency\;\left(BOD\&TDS\right)\left(\%\right)=\left[\left(C_{o}-C_{f}\right)/C_{o}\right]\times 100$$

(2) $$Energy\;consumption\;\left(ENC\right)\left(kWh/m^{3}\right)=\left(U\times I\times t\right)/V$$

(3) $$Electrode\;consumption\left(ELC\right)\left(kg/m^{3}\right)=\left(I\times t\times M\right)/n\times F\times V$$

(4) $$Operating\;cost\;\left(OP\right)\;\left(USD/m^{3}\right)=aENC+bELC$$

**Table 3:** Detailed comparison of electrode geometry.

**Time (min)**	**Electrode**	**Removal efficiency (%)**		**ENC (kWh/m**^**3**^**)**	**ELC (kg/m**^**3**^**)**	**OP (USD/m**^**3**^**)**
		**BOD**	**TDS**			
15	Plane	20	18	1.0937	0.001	0.089
30		26	30	2.187	0.0012	0.177
45		38	41	3.281	0.0013	0.264
60		50	55	4.375	0.0016	0.352
75		61	65	5.468	0.0017	0.440
90		64	69	6.562	0.0019	0.528
105		68	72	7.656	0.002	0.616
**120**		**69**	**78**	**8.75**	**0.0021**	**0.703**
						
15	01 hole	25	28	1.203	0.0012	0.098
30		29	33	2.406	0.0013	0.194
45		40	44	3.609	0.0015	0.291
60		53	60	4.812	0.0017	0.388
75		64	69	6.015	0.0019	0.484
90		68	72	7.218	0.002	0.581
**105**		**74**	**78**	**8.421**	**0.0021**	**0.677**
120		78	83	9.625	0.0024	0.774
						
15	02 hole	28	32	1.421	0.0015	0.116
30		35	40	2.843	0.0017	0.230
45		43	48	4.2656	0.0019	0.344
60		59	68	5.6875	0.002	0.458
75		69	75	7.109	0.0021	0.572
**90**		**74**	**78**	**8.531**	**0.0024**	**0.686**
105		79	83	9.953	0.0026	0.800
120		85	89	11.375	0.0028	0.914
						
15	03 hole	34	38	1.75	0.0019	0.143
30		39	42	3.5	0.002	0.283
45		49	54	5.25	0.0021	0.423
60		61	70	7	0.0024	0.564
**75**		**73**	**79**	**8.75**	**0.0026**	**0.674**
90		82	88	10.5	0.0028	0.844
105		86	90	12.25	0.003	0.985
120		97	98	14	0.0032	1.125
						
15	04 hole	38	42	1.968	0.0021	0.161
30		43	45	3.937	0.0024	0.3192
45		51	58	5.906	0.0026	0.477
**60**		**76**	**80**	**7.875**	**0.0028**	**0.634**
75		77	88	9.843	0.003	0.792
90		88	91	11.812	0.0032	0.950
105		94	94	13.78	0.0034	1.108
120		100	100	15.75	0.0036	1.266

#### 2.3 Characterization of EC sludge

The sludge generated during the electrocoagulation treatment is highly complex in nature. In this context, the characterization of electrocoagulation-generated sludge has been performed for manufacturing of non-constructional building blocks (seen in Fig. 6, Fig. 7).
