# Supplementary material for: Data on treatment of sewage wastewater by electrocoagulation using punched aluminum electrode and characterization of generated sludge
Source: Data Brief. 2018 Apr 11;18:1229–38. doi: 10.1016/j.dib.2018.04.020 (PMC6058665; doi:10.1016/j.dib.2018.04.020)
Supplement: Supplementary file 1 — Supplementary material [file mmc1.pdf]

## Conflict of Interest

*The authors have declared no conflict of interest*


**Corresponding author**

Name: Dr. Vinita Khandegar (Assistant Professor)
Affiliation: University School of Chemical Technology
Guru Gobind Singh Indraprastha University, Dwarka New Delhi-110078, India
